# Absence of renal enlargement in fructose‐fed proximal‐tubule‐select insulin receptor (IR), insulin‐like‐growth factor receptor (IGF1R) double knockout mice

**DOI:** 10.14814/phy2.13052

**Published:** 2016-12-06

**Authors:** Lijun Li, Marcus Byrd, Kwame Doh, Patrice D. Dixon, Hwal Lee, Swasti Tiwari, Carolyn M. Ecelbarger

**Affiliations:** ^1^ Department of MedicineGeorgetown UniversityWashingtonDistrict of Columbia; ^2^ Department of Molecular Medicine & BiotechnologySanjay Gandhi Postgraduate Institute of Medical SciencesLucknowIndia

**Keywords:** Insulin resistance, metabolic syndrome, renal enlargement, renal tubular acidosis, sex differences

## Abstract

The major site of fructose metabolism in the kidney is the proximal tubule (PT). To test whether insulin and/or IGF1 signaling in the PT is involved in renal structural/functional responses to dietary fructose, we bred mice with dual knockout (KO) of the insulin receptor (IR) and the IGF1 receptor (IGF1R) in PT by Cre‐lox recombination, using a *γ*‐glutamyl transferase promoter. KO mice had slightly (~10%) reduced body and kidney weights, as well as, a reduction in mean protein‐to‐DNA ratio in kidney cortex suggesting smaller cell size. Under control diet, IR and IGF1R protein band densities were 30–50% (*P* < 0.05) lower than WT, and the relative difference was greater in male animals. Male, but not female KO, also had significantly reduced band densities for Akt (protein kinase B), phosphorylated Akt^T308^ and IR^Y^ ^1162/1163^. A high‐fructose diet (1‐month) led to a significant increase in kidney weight in WT males (12%), but not in KO males or in either genotype of female mice. Kidney enlargement in the WT males was accompanied by a small, insignificant fall in protein‐to‐DNA ratio, supporting hyperplasia rather than hypertrophy. Fructose feeding of male WT mice led to significantly higher sodium bicarbonate exchanger (NBCe1), sodium hydrogen exchanger (NHE3), sodium phosphate co‐transporter (NaPi‐2), and transforming growth factor‐β (TGF‐β) abundances, as compared to male KO, suggesting elevated transport capacity and an early feature of fibrosis may have accompanied the renal enlargement. Overall, IR and/or IGF1R appear to have a role in PT cell size and enlargement in response to high‐fructose diet.

## Introduction

Metabolic syndrome (MetS) is a common and escalating constellation of clinically associated disorders including dyslipidemia, insulin resistance, visceral adiposity, and hypertension (Isomaa 2003). Recent estimates are that it may affect as many as 32–35% of the United States population (Ogden et al. 2006). Poor diet and increasing rates of obesity are at the root of MetS. One dietary factor which has increased dramatically in the last 3–4 decades is consumption of the monosaccharide, fructose (Bray 2010). Fructose has remarkably alternative whole‐body metabolism, as compared to glucose, and due to its less rate‐limited metabolism, its rapid conversion to triglycerides can be associated with cellular ATP depletion and high circulating levels of uric acid (Johnson et al. 2013). It has been estimated that 60–70% of reabsorbed fructose is taken up by the liver, and undergoes metabolism at this site (Johnson et al. 2010). Another 30–40% appears to be metabolized by kidney, adipose tissue, and other organs.

In the kidney, the proximal tubule (PT) is likely the predominant site of fructose reabsorption and metabolism. The PT expresses fructose transporters, for example, GLUT5 (*slc2a5*) and GLUT9 (*slc2a9*), as well as, ketohexokinase (KHK), the first enzyme in fructose metabolism. Recent studies have shown that renal GLUT5 and KHK protein and/or mRNA levels are increased in mice fed high‐fructose diets; however, these changes can be strain‐ and sex‐specific (Barone et al. 2009; Aoyama et al. 2012; Sharma et al. 2015). In addition to transporting fructose, GLUT9 is a high‐capacity urate transporter, expressed in the kidney distal convoluted tubule, as well as, PT (Doblado and Moley 2009). In our previous study, we showed reduced plasma uric acid levels in mice fed high‐fructose diet for 3 months leading to the possibility that fructose was competing with urate for reabsorption via this transporter (Sharma et al. 2015). High‐dietary fructose has been associated with increased markers of renal fibrosis, oxidative stress, and activity of the PT sodium hydrogen exchanger (NHE3, *slc9a3*), as well as, other alterations that would support renal disease and hypertension (Johnson et al. 2010, 2013; Queiroz‐Leite et al. 2012).

Impaired insulin receptor (IR, *Insr*) signaling is a cardinal component of MetS (Kido et al. 2001). Tissue‐specific knockout (KO) of IR in mice from brain, adipose tissue, liver, heart, or muscle has been demonstrated to result in distinct phenotypes all associated in some way with MetS (Bruning et al. 1998, 2000; Abel et al. 1999; Michael et al. 2000; Guerra et al. 2001). Our own investigations have revealed that mice with IR deleted from renal PT, using γ‐glutamyl transferase promoter‐driven Cre‐recombinase, have elevated semi‐fasting blood glucose levels and elevated renal cortical mRNA expression level and activity of glucose‐6‐phosphatase (G6Pase) (Tiwari et al. 2013). Yet they responded normally to a hyperinsulinemic‐euglycemic clamp, suggesting the defect, as expected, was localized to the kidney. However, interpretation of the actions of insulin in kidney, as well as other organs, is complicated somewhat by similarities to a related hormone, the insulin‐like‐growth factor (IGF).

The IGF type 1 receptor (IGF1R, *Igf1r*) and IR are coupled to related, but not identical, signaling cascades in many cell types. IGF (both I and II) is associated primarily with mitogenic/growth pathways, and has been demonstrated to have a role in tumor biology (Isoyama et al. 2015). In contrast, insulin's actions are primarily metabolic and include uptake of glucose into cells, as well as, down‐regulation of glucose‐production pathways, including gluconeogenesis in liver and likely renal PT (Siddle 2011). However, downstream signaling in both instances is related to activation of phosphoinositide‐3‐kinase (PI‐3K) and protein kinase B (Akt) phosphorylation, and many down‐stream actions of the two hormones have been shown to be similar. Moreover, insulin can bind to IGF1R, and vice versa, although with limited avidity. For example, insulin binds to IGF1R with about 500–1000‐fold lower affinity (Garcia‐Echeverria et al. 2004). However, IR and IGF1R can form heterotetrameric receptors, which appear to bind only IGF (Samani et al. 2007). While IR and IGF1R are expressed along renal tubules (Nakamura et al. 1983; Butlen et al. 1988; Catena et al. 2003), their physiological role in the maintenance of cellular function in the kidney is not clear.

Therefore, the main aim of the current study was to determine whether dual, cell‐select KO of the IR and IGF1R from the renal PT would alter whole‐body metabolic and renal‐specific responses to dietary fructose in mice. These responses were determined in both male and female mice as our previous studies supported, on the whole, increased PT uptake and fructose metabolism in male mice, at the expense of greater downstream, distal tubular effects in the female counterparts (Sharma et al. 2015).

## Materials and Methods

### Knockout mice

The animal procedures described herein were fully approved by the Georgetown University Animal Care and Use Committee (GUACUC), and were carried out in the Georgetown University Department of Comparative Medicine Animal Facility, an USDA and AAALAC (Association for Assessment and Accreditation of Laboratory Animal Care) International approved facility. Mice with dual knockout (KO) of the insulin receptor (IR) and IGF1 receptor (IGF1R) selectively from the proximal tubule were generated at Georgetown University by crossing mice that were homozygously floxed for both genes (bred in our own colony), with mice carrying Cre‐recombinase driven by the *γ*‐glutamyltransferase (*γ*GT) promoter (obtained originally from E.G. Neilson, Vanderbilt University) (Iwano et al. 2002). We recently used the same Cre‐line to selectively delete IR alone from PT (Tiwari et al. 2013) In subsequent generations, KO females were bred back to doubly floxed IR male mice to produce progeny of which about 50% were heterozygous for *γ*GT‐Cre (KO) and 50% were homozygous “wild‐type” for this allele (WT). All offspring were homozygous for floxed IR and IGF1R. KO mice were determined by standard polymerase chain reaction (PCR) genotyping of tail DNA, using previously described primers (Tiwari et al. 2013).

### Study design

To test whether the lack of IR/IGF1R in the PT altered renal PT responses to dietary fructose, adult KO and WT mice were randomized to receive either Purina Lab Diet 5001 (control diet) or a high‐fructose diet (TD.89247, 60% fructose, dry weight, Harlan‐Teklad, Madison, WI) for 4 weeks. Table 1 shows a comparison of dietary constituents. Four separate cohorts (consisting of 4–5 litters each) were studied (total *n* = 11–15 mice/genotype/diet/sex). At 4 weeks, mice were euthanized by exsanguination via the heart under inactin/xylazine anesthesia.

**Table 1:** Approximate composition of diets

Category	Ingredient	Control^a^ (%)	Fructose^b^ (%)
Protein	Mixed Source	25	
	Casein		20.7
	DL‐Methionine		0.3
Carbohydrate	Starch	21	
	Fructose	0.27	60
	Sucrose	3.83	
	Other	18.8	
Fat	Lard (mainly)	6.4	5
Fiber	Cellulose		7.98
	Mixed Source	16.7	
Minerals	Potassium	1.28	0.49
	Sodium	0.39	0.27
	Chloride	0.64	0.63
	Phosphorus (nonphytate)	0.42	0.39
	Other	4.27	3.24
Vitamins		1	1
Total		100	100

a % by weight; Purina 5001 Rodent Chow.

b % by weight; Teklad TD.89247.

### Plasma/blood analyses

In the final week, semi‐fasting blood glucose levels were measured by glucometer on tail blood after 5 h of fasting. After euthanizing, heparinized blood was collected, centrifuged, and plasma obtained for analysis. ELISAs were performed for insulin (Ultra‐Sensitive Mouse ELISA kit, Crystal Chem, Downers Grove, IL) and IGF1 (ab100695, Abcam, Cambridge, UK) according to the manufacturers’ instructions. Triglycerides were determined by a colorimetric assay (BioVision Incorporated, Milpitas, CA).

### Kidney preparation and analyses

After rapid removal, the left kidney was either homogenized whole or dissected into cortex and medulla. Whole‐cell cortex homogenates were prepared by dissecting cortical tissue free from medullary tissue, mincing it, then homogenizing it in a solution containing protease inhibitors in a buffered 250 mmol/L sucrose solution, as previously described (Ecelbarger et al. 2001). In one cohort, the right kidney was perfusion fixed with 4% paraformaldehyde and mounted in paraffin. Masson's trichrome staining of sectioned kidney to assess collagen deposition was performed by the Lombardi Cancer Center Histology Core Facility (Georgetown University). Immunoperoxidase‐based immunohistochemistry for IR, IGF1R, Cre‐recombinase, and proliferating cell nuclear antigen (PCNA) was performed on sectioned kidney as previously described (Tiwari et al. 2013; Sharma et al. 2015). NaPi‐2 (sodium phosphate cotransporter‐2, *slc34a2*) was used as a marker to identify PT. Antibodies used were our own polyclonal rabbit for NaPi‐2 and commercial antibodies for IR (Abcam, ab5500), IGF1R (Santa Cruz, sc713), PCNA (Cell Signaling, D3H8P), and Cre‐recombinase (Sigma‐Aldrich, clone 2D8 monoclonal). DNA and protein concentrations of the cortex homogenates were determined by a fluorescent approach, using bisBenzimide H 33258 (Hoechst 33258) via a DNA Quantitation Kit (DNAQF, Sigma‐Aldrich), and Pierce BCA (bicinchoninic acid) protein assay kit, respectively.

### Western blotting

Western blotting was performed by the methods established in our laboratory (Song et al. 2004a). After determining the protein concentrations of the homogenates, the protein was solubilized in Laemmli sample buffer. Quality of tissue sample preparation was assessed by staining loading gels with Coomassie‐blue (Gelcode Blue, Pierce Endogen, Rockford, IL), and then examining the sharpness and intensity of the bands. To assess the alterations in the protein abundances, semi‐quantitative immunoblotting was performed. For immunoblotting, 10–15 *μ*g of protein from each sample were loaded into individual lanes of minigels of 10 or 12% polyacrylamide (precast, BioRad, Hercules, CA). Blots were probed with our own polyclonal rabbit antibodies against NaPi‐2 and NHE3 designed to the same sequences as reported previously (Fernandez‐Llama et al. 1998; Kwon et al. 1999). Commercial antibodies from Santa Cruz Biotechnology (Dallas, TX) were used for GLUT5 (rabbit polyclonal H200, sc‐30109), IR*β* (sc‐711), p‐tyr1162/1163 IR*β* (sc‐25103), IGF1R*β* (sc‐713), KHK (sc‐366024), and NBCe1 (sc‐162214, electrogenic sodium bicarbonate co‐transporter‐1, *slc4a4*). A commercial polyclonal rabbit antibody from Novus Biologicals (Littleton, CO) was used for GLUT9, and rabbit polyclonals from Cell Signaling Technology (Danvers, MA) were used for p‐thr 308 Akt (9275), Akt (4685), transforming growth factor beta 1 (TGFβ, 3711), and the mammalian target of rapamycin (mTOR, 7C10). Loading accuracy was evaluated by probing the lower portion of nitrocellulose membranes with *β*‐actin mouse monoclonal antibody (Abcam). Blots and band densities were first normalized by *β*‐actin, and then expressed relative to the mean density of the male control WT mean.

### Statistical analysis

Quantitative data are expressed as mean ± SEM. Statistically significant differences due to the main factors (genotype, diet, and sex) and their interactions were determined by 3‐way ANOVA. Unpaired t‐tests were also used to evaluate differences between the two genotypes (within the same sex and diet), and between the two diets (within the same sex and genotype). *P* < 0.05 were considered significant for all tests (Sigma Plot 10.0, Systat Software, Inc., San Jose, CA).

## Results

### IR and IGF1R expression

**Figure 1:**
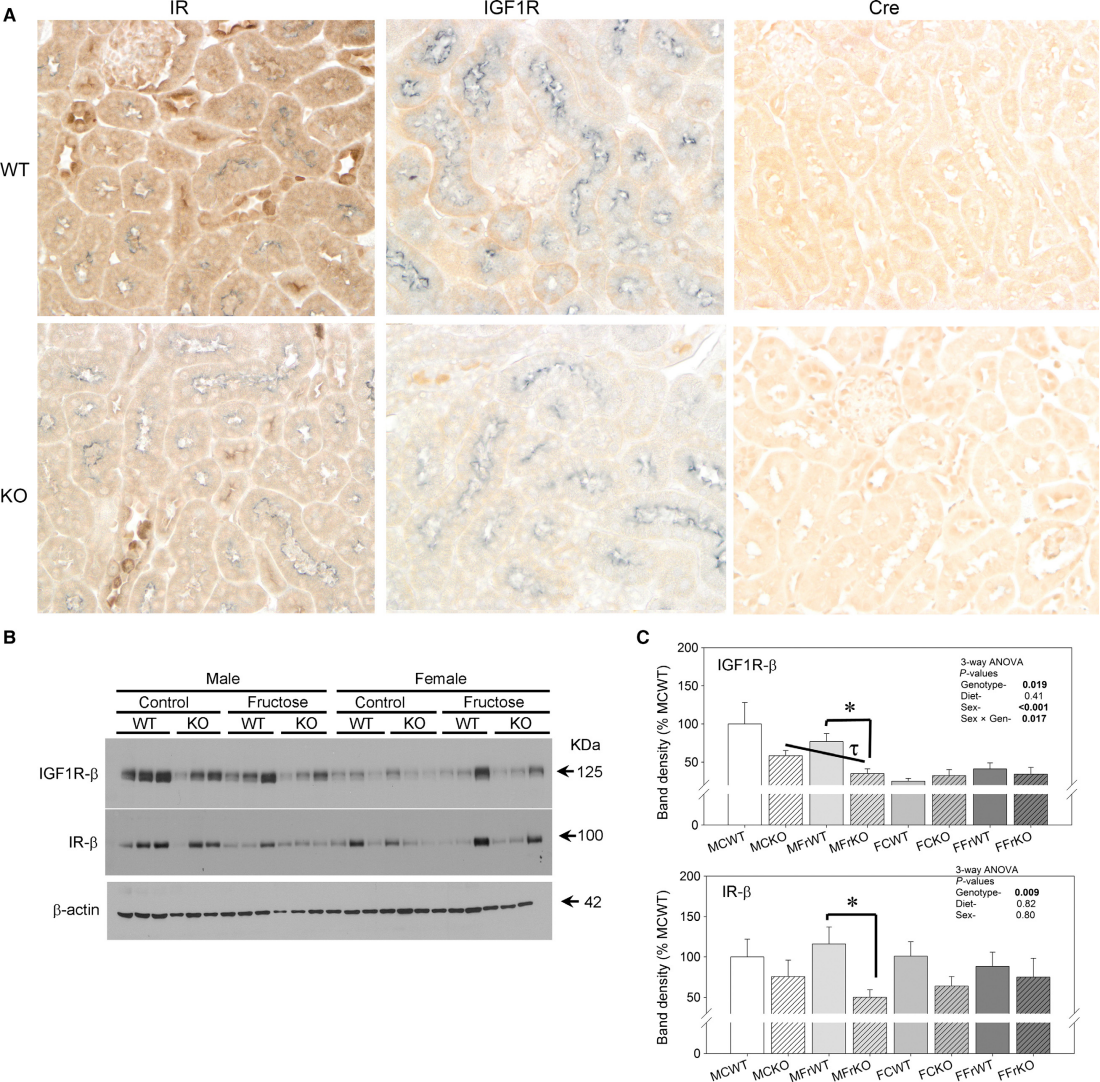
IR/IGF1R double knockout (KO)‐ (A) Immunoperoxidase‐based labeling of IR (brown, left), IGF1R (brown, middle), and Cre‐recombinase (brown, right) in WT (top) and KO (bottom) mice. For first 2 columns, NaPi‐2 (gray) labeling was used to identify proximal tubules. (B) Representative western blots loaded with 20 *μ*g cortex homogenates from mice probed with antibodies against IGF1R (*β*‐subunit) and IR (*β*‐subunit). (C) Densitometry summary‐ 3‐way ANOVA *P*‐values for main factors and significant interactions provided within the figure; band densities were normalized to *β*‐actin and then expressed as %MCWT group. * indicates a significant (*P* < 0.05) difference between genotypes (within same sex and diet) and *τ* indicates a significant difference between diets (within the same genotype and sex) by unpaired t‐test (*n* = 6–9 mice/group).

Figure 1A shows immunohistochemical labeling in cortex for IR, IGF1R, and Cre‐recombinase as indicated. IR labeling was found throughout the tubules with darkest staining in distal tubule apical membranes. IGF1R was predominantly basolateral in proximal tubule, but also strongest in distal tubule sites. Co‐labeling with NaPi‐2 was used to identify proximal tubule. Overall staining was less intense for both IR and IGF1R in proximal tubule in KO mice. KO mice also showed nuclear labeling with Cre‐recombinase, indicating expression of this protein in the expected cells in KO, but not WT mice. Using western blotting (panels B and C), we found on average, a significant 30–50% reduction in band densities for both IR and IGF1R (*β*‐subunits) in the KO, relative to WT, mice, an overall significant genotype effect (3‐way ANOVA). Fructose diet did not result in significant differences in expression. Female mice had reduced IGF1R expression.

### Body and kidney weights

**Figure 2:**
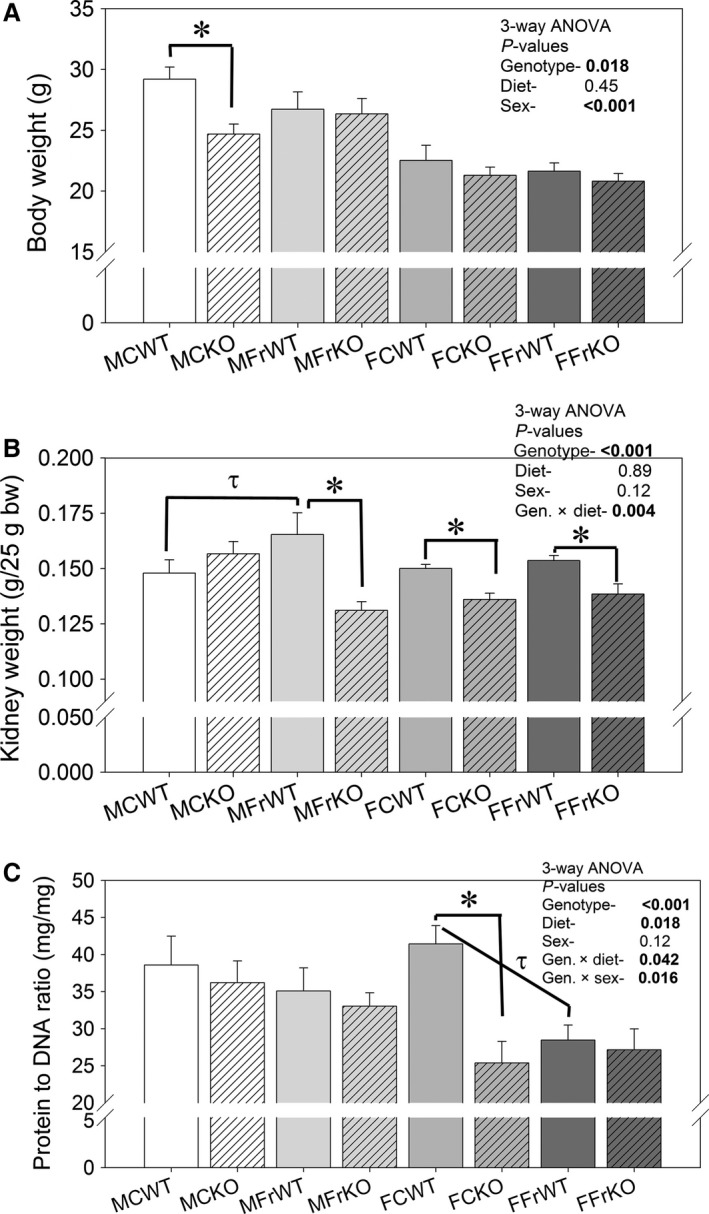
Body and kidney weights‐ (A) Final body weights; (B) final kidney weights (normalized to 25 g body weight) and (C) protein‐to‐DNA ratios in cortex homogenates in control and high‐fructose‐fed mice. Three‐way ANOVA *P*‐values for main factors and significant interactions provided within the figure; * indicates a significant (*P* < 0.05) difference between genotypes (within the same sex and diet) and *τ* indicates a significant difference between diets (within the same genotype and sex) by unpaired t‐test (*n* = 6–9 mice/group).

Body and kidney weights and protein‐to‐DNA ratio in kidney cortex are shown in Figure 2. Three‐way ANOVA revealed that KO mice had modestly, but significantly, lower final body weights (Fig. 2A) and body‐weight‐normalized kidney weights (except in control‐fed males, Fig. 2B). Absolute kidney weights (not shown) were also lower in KO (by 3‐way ANOVA). Diet did not significantly affect kidney weights or body weights, except in WT males, where fructose increased kidney weight so that it was significantly higher than in fructose‐fed KO males and control‐fed WT males. As expected, females weighed less, but body weight normalized kidney weights were not significantly different (than males). Furthermore, female mice of either genotype did not show an increase in kidney weight with fructose‐feeding.

**Figure 3:**
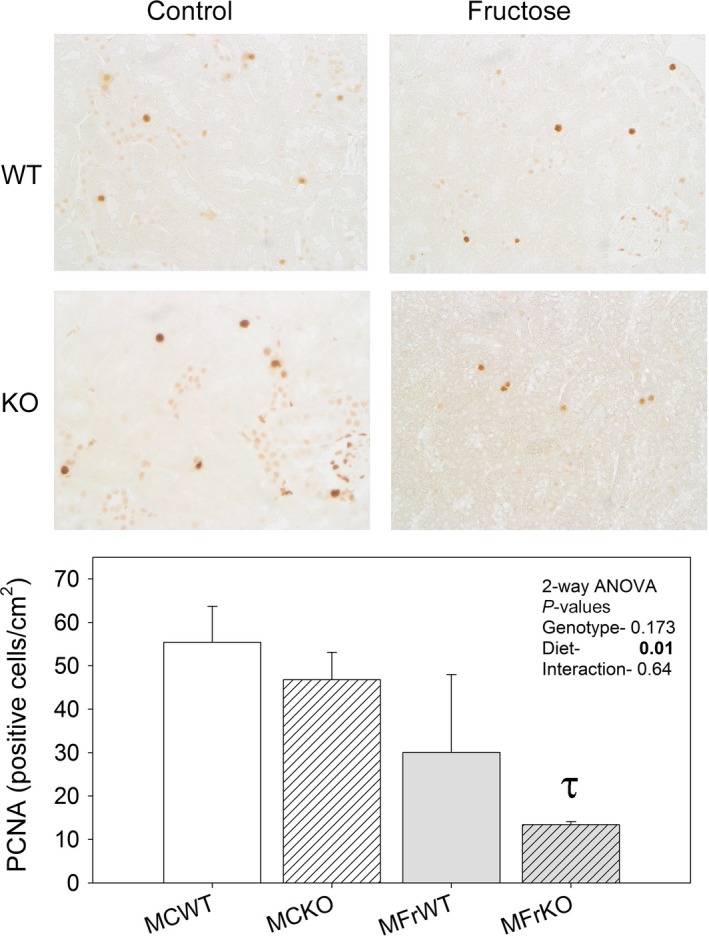
Proliferating cell nuclear antigen (PCNA) labeling‐ Immunoperoxidase‐based labeling of PCNA‐positive cells. Mean ± sem of positive cell counts per cm^2^ tissue slice are shown (*n* = 3 mice/group). *τ* Indicates a significant difference between diets (within the same genotype and sex) by unpaired t‐test.

Protein‐to‐DNA ratio in cortex homogenates (Fig. 2C) was measured as an index of cell size, and used to provide insight as to whether the increased weight of the kidneys in MFrWT mice was due to hypertrophy and/or hyperplasia. KO mice had significantly lower protein‐to‐DNA ratios (3‐way ANOVA, *P* < 0.001 for genotype) indicating the average cell protein level (usually indicative of the cell size) was reduced in cortex homogenates in the KO. This reduction was intensified in females (genotype x sex interaction). The response to fructose was also significant, and showed a reduction in this ratio as well (*P* = 0.018). PCNA staining was conducted on fixed tissue slices from the male mice to evaluate differences in the number/rate of proliferating cells (Fig. 3). In all four groups, we found positive cells; however, surprisingly, fructose diet led to a significant reduction in the density of positive cells, especially in KO mice.

### Plasma circulating factors and hormones

**Figure 4:**
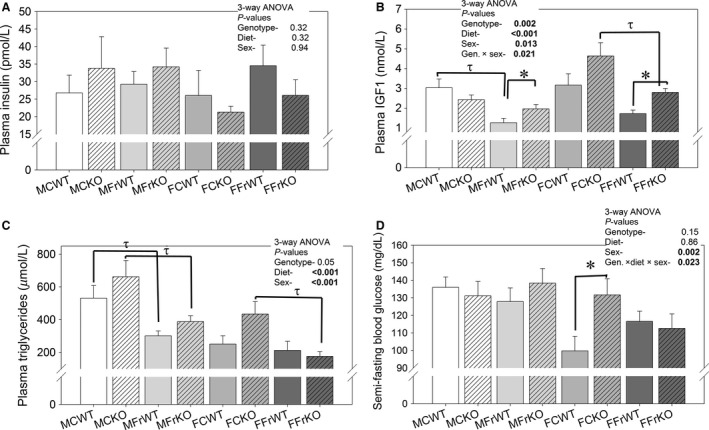
Blood hormones and metabolic determinants‐ **(**A) plasma insulin and (B) plasma IGF1; (C) plasma triglycerides; and (D) semi‐fasting (5‐hour fast) blood glucose concentrations in mice fed control or fructose diet. Three‐way ANOVA p‐values for main factors and significant interactions provided within the figure; * indicates a significant (*P* < 0.05) difference between genotypes (within the same sex and diet) and *τ* indicates a significant difference between diets (within the same genotype and sex) by unpaired t‐test (*n* = 6–9 mice/group).

We next assessed whether circulating factors were differentially regulated amongst the groups. In Figure 4, we show final plasma insulin, IGF1, triglycerides, and semi‐fasting blood glucose. Plasma insulin was not significantly affected by genotype, diet, or sex (panel A, 3‐way ANOVA). Interactions were also insignificant (not shown). In contrast, IGF1 was significantly reduced by fructose, as determined by 3‐way ANOVA (panel B). It was also significantly higher in female mice and in KO mice. There were also a number of significant interactive terms for plasma IGF1. Female and fructose‐fed mice had significantly (3‐way ANOVA) reduced plasma triglycerides (panel C). However, in the WT females, fructose‐feeding had little effect. Finally female mice also had significantly lower semi‐fasting blood glucose (SFBG) levels. However, diet and genotype had inconsistent effects on this parameter (3‐way interaction).

### Collagen deposition

**Figure 5:**
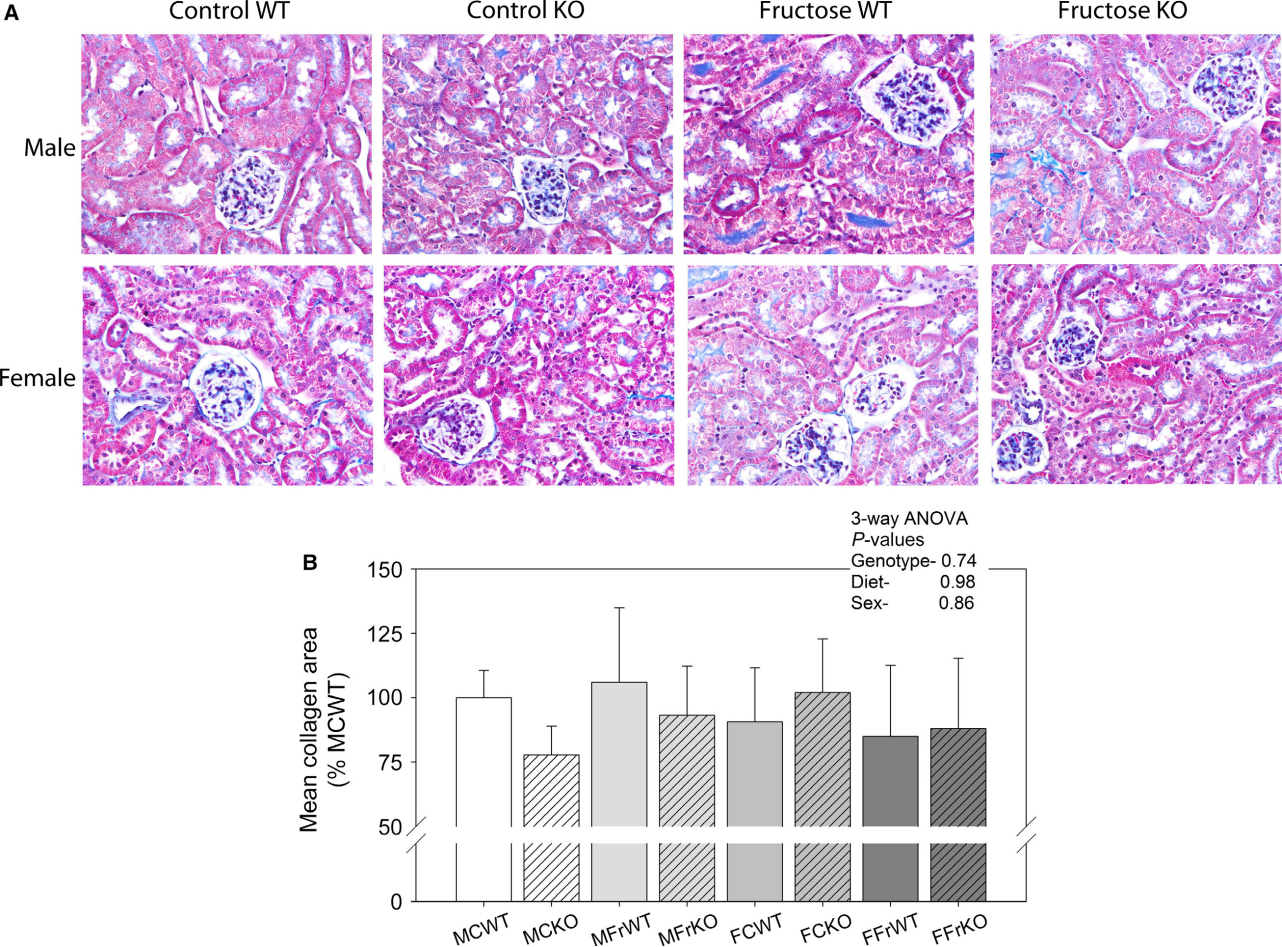
Masson's trichrome staining‐ (A) Representative images from a mouse in each of the eight groups stained with Masson's trichrome to evaluate collagen deposition. (B) Area summary (mean ± SEM); No significant differences between groups or due to major factors (genotype, sex, or diet) were found for area of blue staining (Image J, NIH) with analysis of stained areas (*n* = 3 mice/group). Equivalent numbers of pixels were analyzed for each mouse. Kidney slides were analyzed in a blinded fashion.

Chronic feeding of dietary fructose to rodents has been associated with collagen deposition in the interstitium of the cortex and medulla (Johnson et al. 2010). To determine whether renal enlargement in WT males was associated with increased collagen, we stained fixed sections of kidney with Masson's Trichrome (Figure 5). Fructose feeding for 1 month did not lead to appreciable collagen deposition in the kidney of our mice. Area analysis of blue staining revealed no significant differences between groups.

### Transporter, exchanger, channel alterations

**Figure 6:**
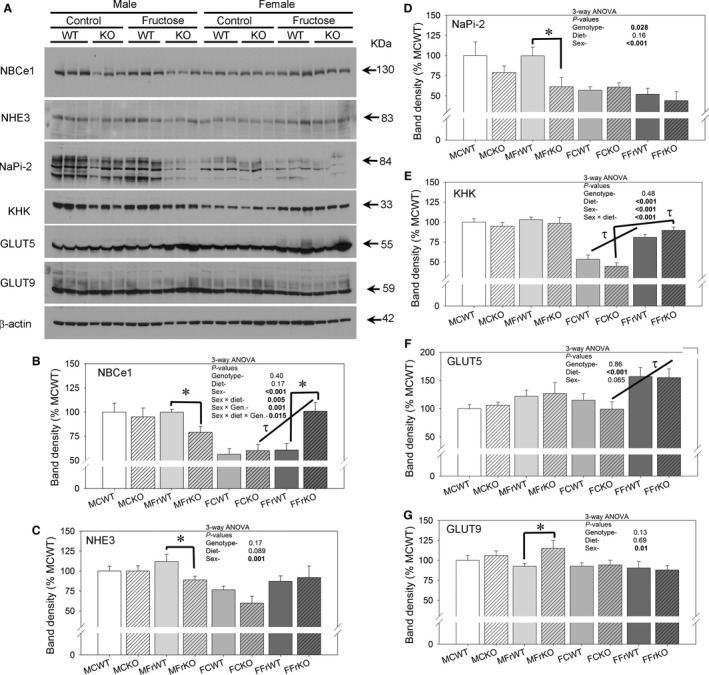
Abundance of sodium and fructose transporters in PT‐ **(**A) Representative western blots of cortex homogenates probed with antibodies against NBCe1, NHE3, NaPi‐2, KHK, GLUT5, GLUT9, and *β*‐actin. Each lane is loaded with the same amount of total protein from three mice each in the different groups as indicated (*n* = 6–9/group for statistics); (B–G) bar graph summaries (mean ± sem) of each protein's densitometry (normalized first to *β*‐actin then to MCWT group); three‐way ANOVA statistics are shown within each graph for main factors and any significant interactions; * indicates a significant (*P* < 0.05) difference between genotypes (with the same sex and diet) and *τ* indicates a significant difference between diets (within the same genotype and sex) by unpaired t‐test.

To determine whether there were differences in the relative abundance or concentrations of major transporters and exchangers in the PT, we conducted western blotting of cortex homogenates. Representative western blots and densitometry summaries are provided in Figure 6. NBCe1, NHE3, and NaPi‐2 concentrations were significantly higher in the MFrWT group as compared to the MFrKO group (panels B‐D). Female mice did not have genotype differences in these transporters except NBCe1, which was higher in the fructose‐fed KO group.

To determine whether potentially elevated transport (uptake) and metabolism of fructose might play a role in renal enlargement, we analyzed the abundances of GLUT5, GLUT9, and KHK (panels E–G). In male mice, no differences were found between genotypes for KHK or GLUT5, while GLUT9 was higher in the MFrKO group (relative to MFrWT). Both KHK and GLUT5 abundances were significantly increased by fructose.

### Insulin/IGF1 receptor‐associated signaling

**Figure 7:**
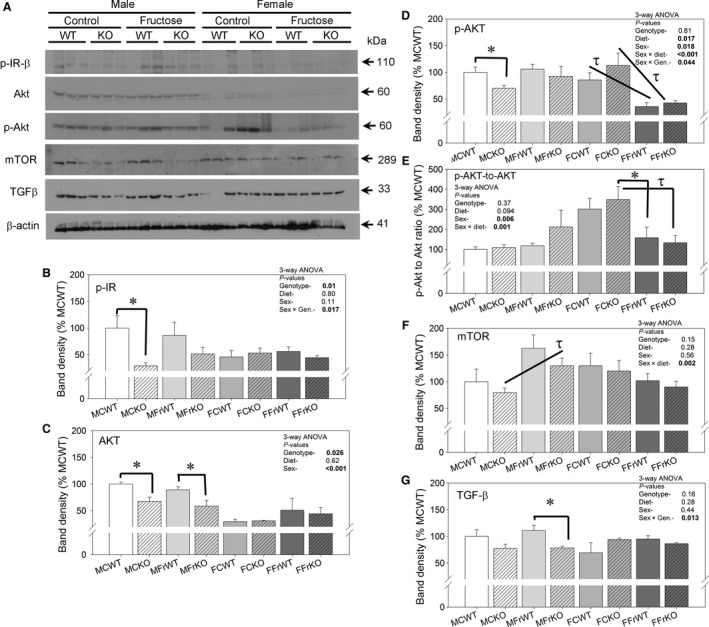
Abundance of insulin/IGF1 signaling components‐ (A) Representative western blots probed with antibodies against p‐IR (Tyr1162/1163), Akt, p‐AKT (Thr308), mTOR, transforming growth factor(TGF)‐*β*, and *β*‐actin. Each lane is loaded with the same amount of total protein from cortex homogenates from three mice each in the different groups as indicated (*n* = 6–9/group for statistics); (B–G) bar graph summaries (mean ± sem) of each protein's densitometry (normalized first to *β*‐actin then to MCWT group); (E) is the ratio of band density for p‐Akt‐to‐Akt (an indicator of relative activity of Akt); three‐way ANOVA statistics are shown within each graph for main factors and any significant interactions; * indicates a significant (*P* < 0.05) difference between genotypes (with the same sex and diet) and *τ* indicates a significant difference between diets (within the same genotype and sex) by unpaired t‐test.

To determine whether differences in early IR/IG1R signaling were apparent amongst groups, western blotting was conducted on various proteins involved in the IR/IGF1R signaling cascade. We also evaluated the expression of TGF‐*β* to determine whether enlargement was associated with increased expression of this pro‐fibrotic cytokine (Figure 7). We found KO mice had significantly reduced phosphorylated IR and AKT concentrations in kidney cortex homogenates (panels B and C). However, this effect was most evident in male mice, that is, there was a significant sex x genotype interaction. Here, mTOR was increased by fructose in KO male mice (panel F), and TGF−*β* abundance was higher in the MFrWT versus the MFrKO mice (panel G). Females had significantly lower Akt band density, as well as, a reduction in p‐Akt with fructose. The ratio of p‐Akt to Akt was highest in control‐fed females and substantially reduced by fructose feeding (panel E). Males showed no difference in this ratio in response to diet.

## Discussion

Impaired insulin receptor (IR) signaling has been associated with a number of pathological conditions including diabetes, hypertension, and renal disease. In the kidney, insulin has been shown to regulate distal tubule sodium reabsorption, as well as, proximal tubule (PT) gluconeogenesis. Less is understood regarding the role of the IGF in the kidneys, although it also appears it may also regulate collecting duct sodium uptake (Gonzalez‐Rodriguez et al. 2007) and gluconeogenesis (Pennisi et al. 2006). Moreover, reduced circulating levels of IGF, such as due to growth hormone deficiency, have been associated with a number of features of the metabolic syndrome (MetS) (Franco et al. 2006). To address the role of IR and IGF1R in the renal PT, we bred mice with PT‐cell select KO of both receptors, using the *γ*‐glutamyl transferase promoter to drive Cre‐recombinase expression in this cell type. Our previous studies (Tiwari et al. 2013) showed that this promoter is specific for renal PT, and does not cause genetic recombination in other candidate sites, for example, liver. Male and female mice were fed standard rodent chow (Purina 5001) or a custom‐formulated high‐fructose diet for 4 weeks.

We found cortical homogenate protein abundances for IR and IGF1R were reduced 30–50%, on average in the KO, with no effect of diet. The cortex homogenate would include several other cell types expressing these receptors, including distal tubule, glomerulus, thick ascending limb, as well as, vascular and interstitial cells. Male WT mice showed a significant (about 12%) increase in the wet weight of the kidney in response to fructose feeding. This response was absent in the male KO mice, as well as, in both genotypes of female mice. These findings will be discussed in greater detail below.

We (Song et al. 2004b; Sharma et al. 2015) and others (Nakayama et al. 2010) have reported that fructose feeding results in renal enlargement in both mice and rats. Tubular hyperplasia, as well as, increased proliferating cell nuclear antigen (PCNA) staining was reported by Nakayama et al. (2010) in the renal cortex and outer stripe of the outer medulla in fructose‐fed rats. This group of investigators also showed an increase in the size of the PT, as well as, a reduction in the nuclear‐to‐epithelial area, which would indicate proliferative hyperplasia. In agreement, we found fructose feeding reduced the protein‐to‐DNA ratio in cortex homogenate; however, we did not find fructose feeding increased the density of PCNA‐positive cells. Different species and length of studies (their study was 6 weeks) may account for some of these differences. In addition, we showed reduced protein‐to‐DNA ratio in the KO mice (both males and females). This may indicate smaller cell size in KO mice cortices. With regard to PCNA staining, we were surprised to see higher levels in the control‐fed mice than in fructose‐fed animals. This reduction was mainly driven by lower density of PCNA‐positive cells in the KO mice fed fructose. This may indicate a role for IR/IGF1R in proliferation of PT. Our data suggest that the differences in kidney weight and responses to dietary fructose were not due to differences in circulating insulin or IGF1. In fact, we found KO mice had higher circulating IGF1. This was intriguing and would suggest that receptor levels in the kidney PT alone may provide feedback to IGF1‐secreting tissues, for example, in the liver in an attempt to boost the signal. This is similar to what is well characterized for insulin in the condition of impaired IR signaling; however, with regard to insulin the signal for the increase is fairly well known in that elevated glucose levels can affect pancreatic insulin production and secretion. The signal for increased IGF1 secretion and/or production in our model system is not known.

In addition, fructose feeding reduced circulating plasma triglyceride levels by about 25%. This is in contrast to expectations, and what we and others have reported for rats (Song et al. 2004b) and humans (Hwang et al. 1987; Sobrecases et al. 2010). However, it has been shown that background strain in mice (as well as rats) is a strong determinant of the cardiovascular and lipid profile effects of fructose feeding (Vrana et al. 2006; Aoyama et al. 2012). Our mice were on a mixed C57Bl6/129/Sv background, and our study was fairly short at 4 weeks. Moreover, half of our study population was female, which is atypical of many studies, using primarily male rodents. We did see a borderline effect of fructose feeding to increase weight gain only in male mice (*P* = 0.059). Somewhat in agreement, Tillman et al. (2014) showed no effect of fructose feeding on triglycerides levels or body weight in C57Bl6 mice fed for 3 months with a high‐fructose diet. Of note, the C57Bl6 strain is relatively susceptible to obesity with general aging and high‐fat feeding (West et al. 1992).

The increase in kidney weight in the WT male mice with fructose feeding was accompanied by an increase in renal expression of the major apical sodium transporters/exchangers: NBCe1, NaPi‐2, and NHE3 (relative to KO males). In agreement, a recent study by Queiroz‐Leite et al. (2012) demonstrated a role for fructose stimulated NHE3 activity in the PT. Thus, the nature of the increase in kidney weight supported a functional adaptive capacity to transport sodium, as opposed to a purely pathological enlargement due to an increase in the expression of collagen or fibrotic proteins. However, we did not find any significant effects of genotype on renal proteins levels of ketohexokinase (KHK), the first enzyme in fructose metabolism, or in GLUT5 (fructose transporter). Male KO mice fed fructose actually had higher expression of GLUT9 (fructose/urate transporter) than did WT males. This would suggest that renal enlargement was not due to relatively higher uptake or metabolism of fructose in the WT males. In an earlier study (Sharma et al. 2015), we did observe higher expression of GLUT5 in the kidney of male, relative to female mice after 3 months of fructose feeding. Our current study, would suggest that females are equally sensitive to dietary fructose with regard to protein abundance changes, at least during the early course of feeding.

We also determined whether we could detect differences in the level of certain markers of IR/IGF1R signaling, using western blotting of cortex. These analyses suggested that baseline IR/IGF1R signaling proteins were expressed at a higher level in male mice, and that reduced signaling was possible in the PT from the KO mice, as a whole, for example, phospho‐Tyr 1161/1162 and AKT (protein kinase B) band densities were reduced in male KO versus male WT kidney. These differences were markedly attenuated in female mice. Taken together, they suggest that the renal enlargement response to fructose requires relatively robust IR/IGF1R signaling, which we found relatively attenuated in both female and KO mice.

In addition, we found an increase in TGF‐*β* protein abundance in the MFrWT versus MFrKO mouse kidney cortex. Previous studies have shown an increase in the expression of this growth factor with dietary fructose, which has the capacity to initiate phenotypic transformation of epithelial cells to mesenchymal cells (Schleicher and Weigert 2000). Our study suggests that IR/IGF1R signaling enhances the expression of TGF1‐*β* in response to dietary fructose. This is in agreement with other work and the concept that there is cross‐talk between IGF‐1 and TGF‐*β* in the cell (Danielpour and Song 2006).

It is unclear whether single KO of IR or IGF1R would produce a different phenotype. We did observe a lack of hyperglycemia in the KO mice, which we had observed earlier in the sole PT‐IR KO mice (Tiwari et al. 2013). However, we did not test the double KO mice with an overnight fast, which may be necessary to clearly observe this phenotype. Additional studies are planned in this regard. The single PT‐IR KO mice were shown to have elevated mRNA levels of glucose‐6‐phosphatase (a rate‐limiting enzyme in gluconeogenesis) and reduced activity of this enzyme.

Overall our studies support IR/IGF1R signaling in determining several phenotypic characteristics of the PT associated with cell size and proliferation. IR/IGF1R signaling appears to play a role in fructose‐induced renal enlargement. This renal growth involves both functional adaptive changes that might increase sodium transport, but also early pathological changes that could ultimately lead to fibrosis.

## Conflict of Interest

None declared.
